# Correction to: Model‐based estimates of tumor growth inhibition metrics are time‐independent: A reply to Mistry

**DOI:** 10.1002/psp4.13209

**Published:** 2024-07-23

Claret, L., Han, K. and Bruno, R. (2017), Model‐based estimates of tumor growth inhibition metrics are time‐independent: A reply to Mistry. CPT Pharmacometrics Syst. Pharmacol., 6: 225–225. https://doi.org/10.1002/psp4.12163

The second author of this paper, K. Han, should be listed as Kelong Han.

